# Capturing autonomy, competence, and relatedness at work: further examining and validating an English language version of the work-related basic need satisfaction scale

**DOI:** 10.3389/fpsyg.2024.1304309

**Published:** 2024-10-18

**Authors:** Paul A. Tiffin, Ray Cabrera, Sarah Dexter-Smith, Anja Van den Broeck

**Affiliations:** ^1^Hull York Medical School, University of York, York, United Kingdom; ^2^Tees, Esk and Wear Valleys NHS Foundation Trust, Darlington, United Kingdom; ^3^KU Leuven, Leuven, Belgium; ^4^North West University, Potchefstroom, South Africa

**Keywords:** work satisfaction, measurement, self-determination theory, W-BNS scale, staff morale, Rasch calibration

## Abstract

Self-Determination Theory (SDT) maintains that the satisfaction of the basic psychological needs for *autonomy*, *competence*, and *relatedness* is associated with optimal individual functioning, including in the workplace. A self-report instrument, the Work-related Basic Need Satisfaction scale (W-BNS), has previously been developed and validated in Dutch and Italian. We aimed to validate an English version of the W-BNS. We also evaluated a bifactor model to assess the extent to which the item responses could be explained by a single, underlying general latent trait. A Rasch calibration was also carried out to assess the extent to which the scores conformed to the assumptions of ‘fundamental measurement’ and could be converted to a common metric. We used data from 141 staff employed by a large UK-based mental health service provider. The postulated three-factor structure provided a good fit to the data. However, a bifactor model, introducing an underlying general factor, provided a superior fit. The items generally conformed to the Rasch measurement model. Evidence of convergent/divergent validity was observed via the correlations between the W-BNS scores and those for the Basic Psychological Needs Satisfaction and Frustration Scale (BPNFS). Regarding construct validity, both the separate needs and total W-BNS scores statistically significantly predicted an individual’s reported intention to leave the current employer. Our findings foster research with the WBNS and have implications for how the W-BNS is optimally implemented in practice as a useful brief tool for assessing staff work-related need satisfaction.

## Introduction

Self-Determination Theory (SDT) postulates three innate (‘basic’) psychological needs which have to be satisfied for individuals to function optimally (Deci and Ryan, 2000). That is, for people to experience well-being, have positive attitudes and display adaptive behaviour (Van den Broeck et al., 2017; Van den Broeck et al., 2016). These needs are: *autonomy* (i.e., a sense that one is generally in control of one’s destiny); *competence* (i.e., a feeling that one is personally effective), and; *relatedness* (i.e., feeling socially connected to others). There is evidence, including a meta-analysis, that having these needs met is associated with positive work related wellbeing and performance (Coxen et al., 2021; Olafsen, 2017; Van den Broeck et al., 2016). Section S1 of the Supplementary Material provides a fuller description of SDT and work-related applications.

Because of its relevance, there have been attempts to develop valid measures of work-related need satisfaction, such as the Work-related Basic Need Satisfaction scale (W-BNS; Van den Broeck et al., 2010). The W-BNS is composed of 18 items, with six items allocated to each of the three ‘basic need’. The instrument uses a five-point Likert scale response format with the labels ‘*strongly agree*’, ‘*somewhat agree*’, ‘*neither agree nor disagree’*, ‘*somewhat disagree*’ and ‘*strongly disagree*’. The psychometric properties were reported to be favourable: the data from the original validation study provided an acceptable fit to a three factor model. Internal reliability consistency was high for all three scales; (i.e., Cronbach’s alpha for *autonomy*, relatedness and competence was 0.82; 0.82, and; 0.91, respectively). In terms of construct validity, *autonomy* satisfaction (though not the other two needs) was inversely, and statistically significantly, associated with staff turn-over. The W-BNS contains both positively (*n* = 10) and negatively (*n* = 8) worded items but there was no evidence of method effects observed in this regard.

The W-BNS was originally validated in Dutch (Van den Broeck et al., 2010) and subsequently piloted in Italian (Colledani et al., 2018) and Turkish-speaking settings (Silman, 2014). However, the English language version of the tool has not yet been validated. This process is vital if the W-BNS is to be used with confidence more widely. In the original W-BNS development study, in addition to three factor models, hierarchical factor models were also tested. However, where a general factor is being modelled, bifactor models are considered more useful in relation to psychometric instrument development, compared to hierarchical second order factor models. This is partly as the specific and general factor loadings are relatively easy to interpret in a bifactor model (Chen et al., 2006). The findings from such an analysis can help guide how the scores from instrument are summarised. Specifically, to what extent responses to the W-BNS should be summarised as three separate scores versus a single summary metric. The application of item response theory (IRT), and, in particular the Rasch model, can also be helpful in understanding the measurement properties of an instrument. This may be appropriate to apply if a single underlying dimension (factor) explains the majority of variance in responses to an instrument. Other assumptions are also made by the Rasch model, notably that all item discrimination values are equal. In this context ‘discrimination’ refers to an item ability to differentiate between test-respondents with differing levels of the trait or ability being evaluated. A Rasch analysis can indicate whether the scores from a scale exhibit ‘simple summed sufficiency’; that is that the total score contains all the necessary information to discriminate between test-takers. Moreover, instruments where responses conform to the Rasch model can have their scores converted to a common, additive unit of measurement (the log-odds unit, or ‘logit’). See Section S2 of the Supplementary Material for a more detailed explanation of the Rasch model.

A large UK National Health Service (NHS) provider was seeking a brief instrument to use in regular organisational checks of staff morale. The W-BNS was desirable as it was developed on the basis of strong theory, supported by empirical evidence, and is the only existing SDT-orientated tool specific to workplace needs satisfaction. Thus, it was decided to pilot the instrument in order to assess its psychometric properties in an English-speaking population before implementation.

The aim of this study was thus to pilot the English version of the W-BNS in order to evaluate its structure, reliability and validity in this setting. This was also an opportunity to evaluate the measurement properties of the instrument in more detail, to guide optimal implementation in practice.

## Methods

### Procedure

All staff employed by the organisation were invited to participate in the online survey which was publicised via an e-newsletter. As an incentive, participants were entered into a lottery with 18 prizes of £50 retail vouchers.

### Measures

The 18 items of the W-BNS (Van den Broeck et al., 2010) were prompted by “*Below, we ask you about the kind of experiences you actually have in your work life*.” Sample items are: ‘*At work, I feel part of a group’* and ‘*I feel competent at my job*’. Answers were coded on a scale from 1 (‘totally disagree’) to 5 (‘totally agree’). The 24 items of the Basic Psychological Needs Satisfaction and Frustration Scale (BPNFS; Van der Kaap-Deeder et al., 2020), which measures SDT-related need satisfaction in general, was included as a measure to evaluate evidence of convergent validity. Sample items are ‘*I feel confident that I can do things well*.’ and ‘*I feel pressured to do too many things*’. Answers were coded on a scale from 1 (‘*Not true at all’*) to 5 (‘*Completely true’*). Respondents were asked about their work intentions and perceived risk of work-stress related sick leave. This information was captured using three bespoke questions designed for this survey using probability response options:

- “*Over the next 6 to 12 months I feel that the chances I will move jobs within the Trust are*: High (70 to 100% likely), Medium (40 to 70%), Low (40% or lower).”
- “*Over the next 6 to 12 months I feel that the chances I will leave the Trust, for reasons other than retirement* (e.g.*, take a job elsewhere*) *are*: High (70 to 100% likely); Medium (40 to 70%); Low (40% or lower).”
- “*Over the next 6 to 12 months I feel that the chances I will need to take sick leave due to work-related stress are:* High (70 to 100% likely); Medium (40 to 70%); Low (40% or lower).”

### Participants

Responses were received from 141 employees. Full demographic details are provided in Table 1. Of the participants, 74% (104) were female, 92% identified as ‘white British’. In this respect the participants were not statistically significantly different (on chi-squared testing) to the organisation’s wider staff population (*p* > 0.05 in all cases). The age profile of the study sample differed somewhat from the staff population in that respondents were less likely to report being under 30 years (7.5% vs. 17. 9%, chi squared = 5.54, df = 1, *p* = 0.02). Conversely, they were more likely to report being over 50 than the wider staff group (50.4% vs. 26.5%, chi squared = 36.89, df = 1, *p* < 0.0001). The distribution of the questionnaire responses are shown in Supplementary Table S1.

**Table 1:** Description of the sample of respondents to the TEWV temperature check survey.

Variable	N	Missing responses [N (%)]
Professional discipline	Allied Health Professional: 16 (11.76%) Nursing: 36 (26.47%) Psychiatry: 12 (8.82%) Psychology: 12 (8.82%) Other: 60 (44.12%)	5 (3.54%)
Gender	Female: 104 (74.29%) Male: 27 (19.29%) Prefer not to say: 7 (5.00%) Prefer to self-identify: 2 (1.43%)	1 (0.71%)
Sexual orientation	‘Heterosexual or straight’: 121 (85.82%) ‘Bisexual’: 1 (0.71%) ‘Gay man’: 3 (2.13%) ‘Gay woman (lesbian)’: 4 (2.84%) Prefer to self-identify: 2 (1.42%) Prefer not to say: 10 (7.09%)	0 (0%)
Self-identified ethnicity	‘White British’ 131 (92.91%) ‘African’: 1 (0.71%) ‘Any other White background’: 3 (2.13%) ‘Any other mixed background’: 2 (1.42%) ‘Irish’: 1 (0.71%) Other: 1 (0.71%) Prefer not to say: 2 (1.42%)	0 (0%)
Religious faith	Christian: 70 (50.00%) No religion: 62 (44.29%) Buddhist: 2 (1.43%) Prefer not to say: 6 (4.29%)	1 (0.71%)
Length of service	1 year or less: 12 (8.57%) 2 to 3 years: 17 (12.14%) 3 to 5 years: 17 (12.14%) 5 to 10 years: 26 (18.57%) More than 10 years: 67 (47.86%) Prefer not to say: 1 (0.71%)	1 (0.71%)
Age	21–30 years: 10 (7.14%) 31–40 years: 22 (15.71%) 41–50 years: 34 (24.29%) 51–65 years: 65 (46.43%) 66 years and above: 2 (1.43%) Prefer not to say: 7 (5.00%)	1 (0.71%)

### Analysis

Regression based analyses were conducted in Stata v17 MP (StataCorp, 2021). Ordinal factor analyses were implemented in Mplus 8.9 (Muthén and Muthén, 2023) using both robust weighted least squares (WLSMV) to derive commonly cited fit indices and full information maximum likelihood (FIML) as the estimation methods. The latter is recognised as a more efficient estimator where indicators are categorical (Muthen et al., 2015). A Rasch calibration (Rasch, 1960) was conducted using Winsteps version 4.01 (Linacre, 2017). A partial credit model was applied to the categorical item responses. In a Rasch analysis reliability can be appraised in several ways. Specifically, the person reliability coefficient relates to the replicability of the ranking of abilities while the person separation index represents the signal to noise ratio and estimates the ability of a test to reliably differentiate different levels of ability within a cohort (Wright and Masters, 1982).

## Results

### Factor analyses

**Figure 1:**
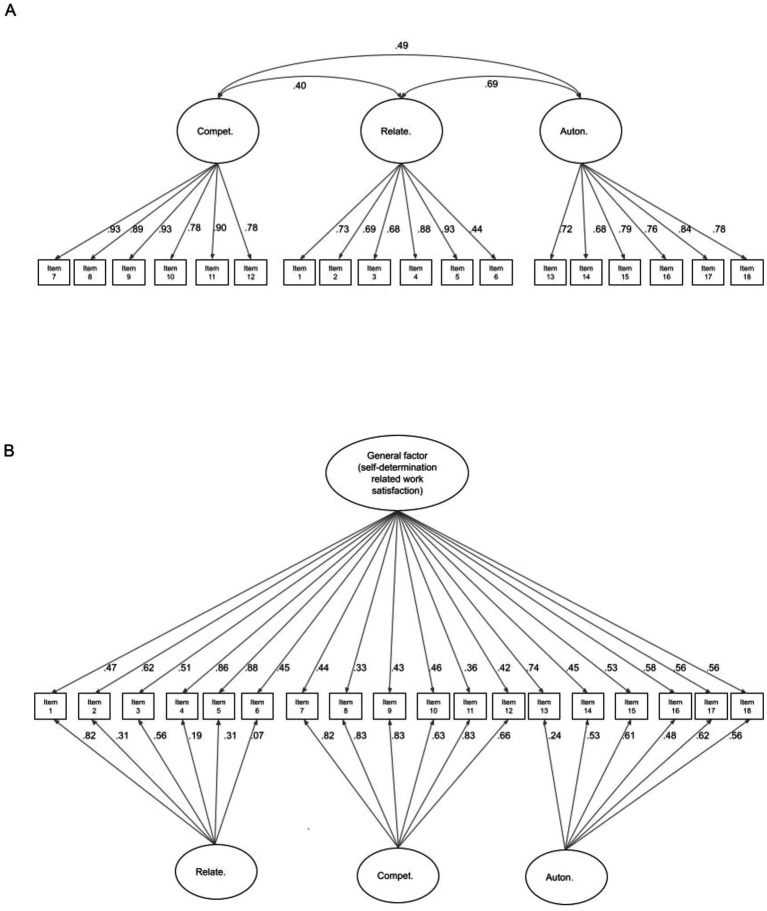
The confirmatory factor analytic (CFA) models with three factors as postulated by the authors of the W-BNS scale **(A)** and the CFA bifactor model **(B)** for the W-BNS response data. Standardised loadings are shown where the variance of the factors are set to 1.

The previously described three factor structure underlying the responses was tested using a confirmatory factor analysis (CFA) model. This demonstrated a good fit to the data (χ^2^ = 319.19, df = 132, *p* < 0.001, Confirmatory Fit Index = 0.96, Tucker-Lewis Fit Index = 0.95, SRMR = 0.066, RSMEA = 0.1). The three factor CFA model is depicted in Figure 1A. Note that the variance of the factors is set to one to derive standardised factor loadings. The modification indices (which suggest model amendments likely to result in significantly improved fit) provided no indication of any significant method effects relating to positively and negatively phrased items. A bifactor model was also fitted (Figure 1B). Again, the variances of the factors were set to 1 to standardise the loadings. Moreover, it should be noted, that to estimate the loading on the general factor, the covariances of the specific factors are constrained to be uncorrelated. The bifactor model could not be estimated via WLSMV so CFI and TLI indices were not available. This may have been due to a combination of the correlation between the underlying factors and the relatively small number of observations. However, the model could be estimated using FIML. Formal chi-squared testing for difference, using the model log likelihoods (Satorra and Bentler, 2010) showed that the bifactor model provided a significantly better fit to the data (χ^2^ = 27.98, df = 15, *p* = 0.02). As can be seen in Figure 1B, all of the items loaded, at least with a magnitude of 0.42 or greater, on the general factor. However, most items also loaded substantially (≥0.3) on a specific factor. The only exceptions to this were for items 4 (“*At work, I can talk with people about things that matter to me*”), 6 (“*Some people I work with are close friends of mine*”) and 13 (“*I feel I can be myself at my job*”).

#### Rasch calibration

Given that all the items loaded substantially on a single, general factor, the unidimensionality assumption seemed plausible. Thus, a Rasch calibration was performed. The concepts of ‘infit’ and ‘outfit’ are unique to Rasch. ‘Fit’ in this sense refers to whether the item responses follow a Guttman sequence (Rasch, 1960). That is, as the ability or trait increases the respondent or test-taker tends to be observed to give a higher scoring category of response, allowing for the play of chance, e.g., 001010111222122122222332333. Items where responses are too predictable ‘overfit’ the model. Those that are more erratic are conceptualised as ‘underfitting.’ The former tends to indicate redundant items that add little information to a scale. In contrast underfitting items can distort or degrade the measurement properties of the scale. ‘Infit’ refers to fit where an item ‘difficulty’ is well matched to the level of trait or ability in a test taker. Conversely, ‘outfit’ refers to fit where item difficulty is not well matched to the test taker’s trait or ability level. The main properties of the W-BNS scale items, in relation to the Rasch calibration, are shown in Table 2. The results indicated that in general the items fitted the Rasch model, with the exception of item 6 (“*Some people I work with are close friends of mine*”) which demonstrated modest underfit. One of the other main item properties, according to the Rasch model, is ‘difficulty’ (often denoted ‘b’). This is sometimes referred to as ‘commonality’ or ‘endorsibility’ if not in relation to ability tests. This relates to the level of trait that a test-taker would have in order to have a particular probability of endorsing a response category. As can be seen from Table 2, a range of ‘endorsibilities’ was observed for the W-BNS items. The item that was most commonly endorsed was item 11 [“*I am good at the things I do in my job*” (b = −0.79)]. The item least commonly endorsed was item 14 (“*I have to follow people’s commands*”), with a difficulty of 1.11 logits.

**Table 2:** W-BNS scale item properties according to a Rasch analysis.

W-BNS scale Item (abbreviated wording)	Difficulty (‘commonality’)	Raw score	Infit (mean-square)	Outfit (mean-square)
1. I do not feel connected with other people	−0.19	479	1.12	1.12
2. At work, I feel part of a group	−0.08	495	1.08	1.09
3. I do not really mix with other people	0.06	463	1.18	1.29
4. At work, I can talk with people	−0.04	473	0.95	0.96
5. I often feel alone	−0.17	502	0.77	0.75
6. Some people I work with are close friends	0.86	381	1.54	1.73
7. I do not really feel competent in my job	−0.82	547	0.86	0.82
8. I really master my tasks at my job	−0.46	501	0.94	0.92
9. I feel competent at my job	−0.65	521	0.82	0.82
10. I doubt I am able to [do] my job properly	−0.52	492	1.03	1.06
11. I am good at the things I do in my job	−0.79	549	0.87	0.80
12. I can accomplish the most difficult tasks	−0.14	498	0.90	0.82
13. I feel like I can be myself at my job	−0.03	470	0.87	0.85
14. I have to follow people’s commands	1.11	381	1.12	1.21
15. I would do things at work differently	0.87	373	0.94	0.98
16. Tasks are in line with what I want to do	0.27	427	0.99	0.99
17. I feel free to do my job the way I think	0.39	436	0.89	0.85
18. In my job I feel forced to do things	0.34	423	0.99	0.99

The Rasch person separation index was 2.88, suggesting that almost three strata of respondents could be differentiated with acceptable precision. As a special case of IRT, Rasch analysis also yields test information curves. These indicate where, in relation to the trait or ability under evaluation, the scale yields most information on each test-taker. In the case of the W-BNS the test information is maximal around the average level of trait in test takers. In this case the trait can be considered as “perceived, general psychological need satisfaction for self-determination in the workplace.”

#### Convergent and divergent validity

In terms of evidence for convergent validity, the correlation matrix (Supplementary Table S2) showed the highest correlations occurred between scores from the two instruments (W-BNS and BPNFS) that related to the same needs. For example, the correlation (r) between the W-BNS and the BPNFS *competence* scores was 0.91. In terms of divergent (‘discriminant’) validity the correlation between different domains were much lower. For example, the W-BNS *relatedness* and BPNFS *competence* scores correlated only 0.37.

#### Construct validity

All the W-BNS scale scores, and the totalled average scores across the needs, statistically significantly predicted a self-reported high probability (“70 to 100%” vs. lower category) of imminently leaving the employer, seeking a different role within the organisation or having to take sick leave (see Table 3). The mean *autonomy* score was most closely associated with these outcomes, especially the perceived risk of imminent sick leave. In this context we treated the W-BNS autonomy score as a ‘screening test” for a self-reported risk of sick leave (see Section S3; Supplementary Figure S1).

**Table 3:** Results of a logistic regression predicting adverse perceived risks (self-reporting 70–100% probability vs. less) from the W-BNS scores, including the total (summed) mean scale scores.

	Odds ratio (OR)	Lower 95% confidence interval for OR	Upper 95% confidence interval for OR	Associated p value
Intention to leave employer				
Autonomy	0.31	0.17	0.56	<0.001
Relatedness	0.61	0.38	0.97	0.04
Competence	0.55	0.34	0.89	0.016
Total mean score	0.32	0.16	0.61	<0.001
Intention to move jobs				
Autonomy	0.27	0.14	0.53	<0.001
Relatedness	0.38	0.22	0.66	<0.001
Competence	0.39	0.23	0.66	<0.001
Total mean score	0.17	0.07	0.39	<0.001
Perceived risk of imminent sick leave				
Autonomy	0.19	0.08	0.42	<0.001
Relatedness	0.54	0.31	0.94	0.028
Competence	0.28	0.15	0.51	<0.001
Total mean score	0.15	0.06	0.37	<0.001

## Discussion

This is the first study to provide evidence of the validity of the English language W-BNS. Our findings replicated those reported for the original validation study (Van den Broeck et al., 2010). Specifically, we showed the postulated three-factor structure provided a good fit to the response data and that the scales demonstrated high internal-consistency reliability. As with the original study, we also observed no significant method effects relating to positively (satisfaction) and negatively (frustration) items. Moreover, we also demonstrated that W-BNS responses can also be considered as relating to a general factor, relating to the total, or averaged, score from all items. In addition to the modestly improved fit of the bifactor model, the general conformity of the items to the Rasch model support that the scale adheres to the principles of ‘fundamental measurement’. That is, the total score should demonstrate ‘simple summed sufficiency’ (i.e., containing all the information required to estimate the respondent’s trait level). This also implies a metric in common units (logits—‘log odds units’) for the W-BNS total score can be created as an overall summary score of ‘work-related satisfaction’. However, the fact that most items also loaded on a specific factor, as well as a general one, suggests that the response structure to the W-BNS is somewhat fractal in nature. That is, while there is a general dimension of self-determination needs-related work satisfaction there are also the three specific facets of *autonomy*, *relatedness* and *competency* within that. This suggests that the total or averaged W-BNS score is a reasonable summary measure of perceived self-determination needs-related work satisfaction. However, capturing and summarising the scores from the three specific scales of the W-BNS is likely to provide additional, more detailed, ‘diagnostic’ information on employee work satisfaction.

In terms of limitations, the evidence for predictive (or construct) validity in the present study is derived from self-report career intentions and perceived risk of sick leave, rather than objective occupational data related to these outcomes. Also, while the staff sample was relatively small it appeared demographically representative in terms of gender and ethnicity of the organisation’s employees. However, respondents, were, on average, significantly older than the general staff pool. However, this should not have adversely impacted the key findings.

Future research could focus on whether employers find the W-BNS useful for both rapid “temperature checks” (evaluations of staff morale) and diagnosing specific issues in the workforce that may be related to one or more of the needs domains assessed. In particular, as in the original validation study, we noted that the *autonomy* score was most sensitive to adverse work outcomes (Van den Broeck et al., 2010). Thus, the *autonomy* scale could act as a rapid screening for risks of adverse occupational events. It may also be possible to create a very short W-BNS scale which correlates highly with the total score, by selected a small number of items that load heavily across the three domains.

## Data Availability

The raw data supporting the conclusions of this article will be made available by the authors, without undue reservation.
