# Supplementary material for: Capturing autonomy, competence, and relatedness at work: further examining and validating an English language version of the work-related basic need satisfaction scale
Source: Front Psychol. 2024 Oct 18;15:1304309. doi: 10.3389/fpsyg.2024.1304309 (PMC11528707; doi:10.3389/fpsyg.2024.1304309)
Supplement: Supplementary file 1 [file Table_1.docx]

**Supplementary material for: Brief Research Report- Capturing autonomy, competence, and relatedness at work: Further examining and validating an English Language version of the Work-related Basic Need Satisfaction (WBNS) scale**

Paul A. Tiffin^1*^, Ray Cabrera^2^, Sarah Dexter-Smith^2^ & Anja Van den Broeck^3^

1. University of York & Hull York Medical School, UK.
2. Tees, Esk and Wear Valleys NHS Foundation Trust.
3. KU Leuven, Belgium; North West University, South-Africa

* Corresponding author. Contact details: Mental Health and Addiction Research Group, Department of Health Sciences, University of York, Area 4, A/B/309, Alcuin Research Resource Centre, University of York, Heslington, York, YO10 5DD, UK. Email: paul.tiffin@york.ac.uk

**Introduction**

This Supplementary Material document contains additional results and some more detailed background information in relation to the Brief Research Report.

**Section S1: Description of Self-Determination Theory and rationale for the study**

Self-Determination Theory (SDT) postulates that there are three innate psychological needs. It is theorised that these have to be satisfied for individuals to function optimally (Deci & Ryan, 2000; Deci, Ryan, & Vansteenkiste, 2008). These are the need for: *autonomy* (i.e. a sense that one is generally in control of one’s destiny; *competence* (i.e. a feeling that one is personally effective), and; *relatedness* (i.e. feeling socially connected to others). There is evidence that an experience of having these needs met is associated with positive outcomes in terms of both wellbeing and functioning (Olafsen, 2017; Van den Broeck et al., 2016; Vansteenkiste, Soenens, & Ryan, 2023). This theoretical framework can also be applied to the workplace (Van den Broeck et al., 2016). Indeed, there is evidence, including one meta-analytic study, that the meeting (or frustration) of these SDT-defined needs is also related to work-related satisfaction and effectiveness (Coxen et al., 2021; Van den Broeck et al., 2017; Van den Broeck et al., 2016). Thus, SDT is likely to be a useful framework for understanding staff satisfaction in a workplace setting for three main reasons:

1. SDT is already a well-established theoretical framework which outlines three distinct, but related, areas related to life satisfaction. These could be potentially translated and applied to a workplace setting.

2. The distinctness of these areas of SDT-orientated need has been supported by prior empirical evidence, e.g. via factor analytic studies. The measurement of the extent to which an employee feels that these needs are met, or unmet, could yield valuable information to organisations about staff morale.

3. SDT thus provides a strong framework to guide workforce policy and interventions related to employee work satisfaction. These could be potentially tailored to the area or areas of need perceived as most satisfied or frustrated.

These reasons have inspired attempts to develop measures that specifically capture work-related satisfaction from an SDT perspective, such as the Work-related Basic Need Satisfaction scale (W-BNS) (Van den Broeck et al., 2010).

**Section S2: Description of the Rasch model and approach to item response modelling**

Item response theory (IRT) is an approach to psychometrics that conceptualises responses to psychological assessments in a probabilistic manner. That is, the particular response observed for a particular test-taker to a specific item is postulated to be due to chance, the level of the trait or ability in the respondent under evaluation, and the particular qualities of that item. In the latter case different IRT models make different assumptions about item qualities. For example, the three parameter IRT model assumes that each item has a certain propensity to be correctly guessed (in the care of knowledge or ability tests), a certain difficulty (that is candidates who are higher on the trait or ability are more likely to endorse, or correctly answer) and a specific discrimination quality (the ability of an item to discriminate between test-takers with different levels of the trait or ability under examination. These traits or abilities are postulated to be (usually normally distributed) latent traits, that cannot be observed or measured directly, only via the observed responses to psychological test items. For a fairly non-technical introduction to IRT please see Livingston, 2020 (Livingston, 2020).

The Rasch model is a special case of an IRT model in that that items are only postulated to have one parameter- ‘difficulty’ (Rasch, 1960). Thus, the Rasch approach conceptualises the probability (or odds) of observing a particular response category from a specific test-taker as due only to chance, the difficulty of the item and the ability (or trait level) of the test-taker. Of course, when considering ability or knowledge tests the term ‘item difficulty’ (or ‘facility’) makes sense. However, IRT is also applied to psychological tests evaluating attitudes or other qualities, such as personality traits. In these cases the term item ‘difficulty’ if often replaced with ‘commonality’ or ‘endorsibility’. For example, a questionnaire may measure political views on a dimension of ‘conservative’ to ‘liberal’. Some items (e.g. “I believe all illegal drugs should be decriminalised’) may only be frequently observed to be endorsed by test-takers high on the trait of ‘liberalism’.

The Rasch model makes strong assumptions; mainly that a scale or test evaluates only a single underlying dimension (factor). The other key assumption made by the Rasch model, is that all items in a scale have the same discrimination values. However, these strong assumptions, if at least approximately fulfilled, give the Rasch model unique qualities not shared by more complex IRT models. For example, a Rasch analysis can indicate whether the scores from a scale exhibit ‘simple summed sufficiency’; that is that the total score contains all the necessary information to discriminate between test-takers. Moreover, instruments where responses conform to the Rasch model can have their scores converted to a common, additive unit of measurement (the log-odds unit, or ‘logit’). The concepts of ‘infit’ and ‘outfit’ are unique to Rasch. ‘Fit’ in this sense refers to whether the item responses follow a Guttman sequence That is, as the ability or trait increases the respondent or test-taker tends to be observed to give a higher scoring category of response, allowing for the play of chance, e.g.,001010111222122122222332333. Items where responses are too predictable ‘overfit’ the model. Those that are more erratic are conceptualised as ‘underfitting.’ The former tends to indicate redundant items that add little information to a scale. In contrast underfitting items can distort or degrade the measurement properties of the scale. ‘Infit’ refers to fit where an item ‘difficulty’ is well matched to the level of trait or ability in a test taker. Conversely, ‘outfit’ refers to fit where item difficulty is not well matched to the test taker’s trait or ability level. Importantly, scales where the items conform to the assumptions and fit expected from the Rasch model are said to provide scores which conform to the tenets of ‘fundamental measurement’. In this sense ‘fundamental measurement’ involves the construction of an additive numerical representation of a unitary (single) dimensional concept that is in a common metric, i.e. three ‘units’ are one more than two ‘units’ (Resse, 1943). Prior to Rasch’s work this was something present in many branches of science, but notably absent from including psychology (Wright, 1999).

As a special case of Item Response Theory (IRT) Rasch analysis also yields test information curves. These indicate where, in relation to the trait or ability under evaluation, the scale yields most information on each test-taker. This is more informative than a single ‘reliability’ index, and recognises that psychological measures resemble thermometers, rather than rulers. That is, a psychological assessment, like a thermometer for temperature measurements, will vary in its accuracy across the range of test-taker traits or abilities.

**Section S3: Supplementary results**

*Distribution of the summary scores for the W-BNS Scales*

| **Scale** | **Mean (SD)** | **Median (range)** | **P for skewness** | **P for kurtosis** |
| --- | --- | --- | --- | --- |
| Autonomy | 3.01 (0.83) | 3 (1.17 to 4.83) | 0.7084 | 0.0035 |
| Relatedness | 3.35(0.85) | 3.5 (1 to 5) | 0.0532 | 0.6865 |
| Competence | 3.74 (0.81) | 4 (1 to 5) | 0.0001 | 0.1613 |

Table S1. Summary statistics for the mean scores derived for the responses to the items for the three scales of the Work (W-BNS)(*autonomy*, *relatedness* and *competence*). Note “P for skewness/kurtosis” denotes the p value for a test for normality, with a p<0.05 indicating statistically significant skewness or kurtosis respectively.

*Convergent and divergent (discriminant) validity*

The scores from the W-BNS correlated with those from the equivalent domains of the Basic Psychological Need Satisfaction and Frustration Scale (BPNFS). The BPNFS measures similar constructs to the W-BNS but the wording of the items is not specific to a work context. From the correlation matrix (Table S2) the highest correlations occurred between scores from the two instruments that report to relate to the same needs. For example, the correlation (r) between the W-BNS and the BPNFS *competence* scores was 0.91. In contrast the magnitude of the observed correlations between different domains on the two instruments were lower. For example, the correlation between the W-BNS *relatedness* and the BPNFS *competence* scores was 0.37.

|  | **W-BNS Autonomy** | **W-BNS Relatedness** | **W-BNS Competence** | **BPNFS Autonomy** | **BPNFS Relatedness** |
| --- | --- | --- | --- | --- | --- |
| **W-BNS Relatedness** | 0.60 |  |  |  |  |
| **W-BNS Competence** | 0.47 | 0.42 |  |  |  |
| **BPNFS Autonomy** | **0.89** | 0.58 | 0.45 |  |  |
| **BPNFS Relatedness** | 0.63 | **0.74** | 0.50 | 0.63 |  |
| **BPNFS Competence** | 0.45 | 0.37 | **0.91** | 0.45 | 0.50 |

Table S2. Correlation between the average scale scores of the W-BNS and the Basic Psychological Need Satisfaction and Frustration Scale (BPNFS) scales (correlations with the equivalent domain scores shown in **bold**).

*The W-BNS autonomy scores as a potential screening test*

According to the present findings and the original validation study (Van den Broeck et al., 2010) the *autonomy* scale score is most closely associated with the risk of adverse work outcomes such as staff turnover (if too high) and perceived risk of imminent sick leave due to work related stress. With this in mind, a receiver operator characteristic (ROC) curve was generated for the ability of the mean autonomy score to predict those respondents who estimated a high (>70%) risk of imminent sick leave. This, in effect plots the ability of the autonomy mean score to identify ‘true positives’ (those reporting a high risk of sick leave) while minimising ‘false positives’ (those with a low autonomy score but who do not report a high risk of sick leave). The ROC curve performs this for different hypothetical “screening” score cut-offs. The area under the curve (AUC) can then be calculated, with 0.5 indicating a test no better than chance and 1.0 reflective of a perfect test. Note, in this case AUCs usually range from 0.5 to 1.0 so the outcome coding was reversed (with a positive case coded as ‘low perceived risk of sick leave’ to prevent a value less than 0.5. The ROC curve is shown in Figure S1, and the associated AUC was estimated to be 0.81.

Figure S1. The receiver operator characteristic (ROC) curve for the average autonomy score of the W-BNS as a screening test for high perceived risk of imminent work-related sick leave.

**References**

Coxen, L., van der Vaart, L., Van den Broeck, A., & Rothmann, S. (2021). Basic Psychological Needs in the Work Context: A Systematic Literature Review of Diary Studies. *Frontiers in Psychology*, *12*, 698526. https://doi.org/10.3389/fpsyg.2021.698526

Deci, E. L., & Ryan, R. M. (2000). The "What" and "Why" of Goal Pursuits: Human Needs and the Self-Determination of Behavior. *Psychological Inquiry*, *11*(4), 227-268. https://doi.org/10.1207/S15327965PLI1104_01

Deci, E. L., Ryan, R. M., & Vansteenkiste, M. (2008). Self-determination theory and the explanatory role of psychological needs in human well-being. In. Oxford University Press.

Livingston, S. A. (2020). *Research Memorandum ETS RM–20-06: Basic Concepts of Item Response Theory: A Nonmathematical Introduction*. T. E. T. Service. https://files.eric.ed.gov/fulltext/ED614352.pdf

Olafsen, A. H. (2017). The implications of need-satisfying work climates on state mindfulness in a longitudinal analysis of work outcomes. *Motivation and Emotion*, *41*(1), 22-37.

Rasch, G. (1960). *Probabilistic models for some intelligence and attainment tests*. The University of Chicago Press.

Resse, T. W. (1943). The application of the theory of physical measurement to the measurement of psychological magnitudes, with three experimental examples. *Psychological Monographs*, *55*(3), i-89. https://doi.org/10.1037/h0093539

Van den Broeck, A., Carpini, J., Leroy, H., & Diefendorff, J. M. (2017). How Much Effort Will I Put into My Work? It Depends on Your Type of Motivation. In *An Introduction to Work and Organizational Psychology* (pp. 354-372). https://doi.org/https://doi.org/10.1002/9781119168058.ch19

Van den Broeck, A., Ferris, D. L., Chang, C.-H., & Rosen, C. C. (2016). A review of self-determination theory’s basic psychological needs at work. *Journal of Management*, *42*(5), 1195-1229.

Van den Broeck, A., Vansteenkiste, M., De Witte, H., Soenens, B., & Lens, W. (2010). Capturing autonomy, competence, and relatedness at work: Construction and initial validation of the Work-related Basic Need Satisfaction scale. *Journal of Occupational and Organizational Psychology*, *83*(4), 981-1002. https://doi.org/https://doi.org/10.1348/096317909X481382

Vansteenkiste, M., Soenens, B., & Ryan, R. M. (2023). 84Basic Psychological Needs Theory: A Conceptual and Empirical Review of Key Criteria. In R. M. Ryan (Ed.), *The Oxford Handbook of Self-Determination Theory* (pp. 0). Oxford University Press. https://doi.org/10.1093/oxfordhb/9780197600047.013.5

Wright, B. D. (1999). Fundamental measurement for psychology. In *The new rules of measurement: What every psychologist and educator should know.* (pp. 65-104). Lawrence Erlbaum Associates Publishers. https://doi.org/10.4324/9781410603593
